# Implementation of a medication adherence program in senior public housing facilities utilizing pharmacists and health educators

**DOI:** 10.17352/2455-5479.000115

**Published:** 2020-11-05

**Authors:** Kimberly Pounds, Dominique Guinn, Ivy O Poon, Aisha Morris Moultry

**Affiliations:** 1 Division of Health Sciences, Health Administration at Texas Southern University, College of Pharmacy and Health Sciences, 3100 Cleburne St., Houston, TX 77004, USA; 2 Department of Health and Kinesiology at Texas Southern University, College of Education, 3100 Cleburne St., Houston, TX 77004, USA; 3 Department of Pharmacy Practice at Texas Southern University, College of Pharmacy and Health Sciences, 3100 Cleburne, Houston TX 77004, USA; 4 Department of Pharmacy Practice and Administration and Associate Dean of Academic Affairs at Texas Southern University, College of Pharmacy and Health Sciences, 3100 Cleburne St., Houston, TX 77004, USA

**Keywords:** Medication adherence, Program implementation, Interdisciplinary care, Behavior change

## Abstract

**Background:**

Hypertension and diabetes disproportionately impact people of color when compared to majority populations. Medication adherence among seniors with chronic diseases has been suboptimal with the estimation that only half of those taking antihypertensives are adherent. Therefore, the purpose of The Managing Your Medications (MY Rx) program was to evaluate the effectiveness of evidence-based practices used to improve rates of medication adherence through information dissemination among diabetic and hypertensive African American, Asian American, and Hispanic residents housed in senior public housing facilities in the Greater Houston Area. The program comprised an 8-week intervention with individual and group components with small incentives provided throughout the program. Individual components included one home visit and telephone consultations conducted by pharmacists. Health educators provided two group education sessions on lifestyle modifications.

**Result:**

Qualitative analysis of focus group discussions revealed participant satisfaction with the MY Rx program and willingness to change after participation in the program.

**Conclusion:**

The Rx program showed the potential effectiveness of an innovative strategy in medication counseling using interdisciplinary pharmacists and health educators to promote health. It demonstrated the importance of using the patient-centered care framework in designing a community intervention program.

## Introduction

People of color are disproportionately burdened by hypertension and diabetes when compared to whites. Hypertension impacts 45% of the adult population, being most prevalent in non-Hispanic African American adults (54%), non-Hispanic white adults (46%), Asians (39%), and Hispanics (36%) [1]. Diabetes impacts 10.5% of the adult population and is greatest among people of color, including Asians (9.2%), Hispanics (12.5%), and African Americans (11.7%) compared to non-Hispanic whites (7.5%) [2]. Adults aged 65 and older make up 10.9% of Houston, TX with 43.7% Hispanic, 20% African American, and 7.3% Asian Americans [3]. Given the likelihood of multiple chronic disease states in these populations and increased risk for reduced medication adherence, there is a critical need to improve medication management to prevent medication-related problems [4].

The Patient-Centered Primary Care Collaborative suggests there is a need for integrating comprehensive medication management within the Patient-Centered Medical Home (PCMH) [5,6]. Pharmacists were identified as healthcare professionals qualified and eff ective in providing medication therapy management services (MTM) to improve prescription medication adherence [7,8]. Additionally, health education strategies are effective in disease state management [9]. Utilizing teams of pharmacists and health educators, the Managing Your Medications (MY Rx) program was developed to evaluate the effectiveness of evidence-based practices used to improve medication adherence behaviors through information dissemination among diabetic and hypertensive African American, Asian American, and Hispanic residents housed in senior public housing facilities in the Greater Houston Area.

### Study design

A quasi-experimental pre- post-study design was used to implement the 12-week My Rx program. Teams of thirteen pharmacists and four health educators were matched to participants based on cultural/ethnic similarity and language. Baseline and follow-up assessments included: blood pressure and/or A1C screening, knowledge of hypertension and/ or diabetes, and behavioral intention related to medication adherence. A thematic analysis of participants program satisfaction was obtained through focus groups at each facility [10]. The study protocol was approved by the Texas Southern University IRB board before the implementation.

The My Rx program took place in four senior public housing facilities operated by the Houston Housing Authority (HHA). Inclusion criteria were age 55 and older, self-identification as either African American, Asian, or Hispanic, resident of the targeted facilities, access to a telephone, and taking at least one medication for diabetes and/ or hypertension at the time of recruitment.

### Description of the intervention development and implementation

A diverse workgroup guided program development through monthly meetings. Membership included nine residence council members (3 individuals/facility), three HHA managers (1/facility), three coalition members (1/coalition), and four My Rx staff.

A list of evidenced-based strategies was provided to the workgroup for consideration. A decision was made through a consensus building process to implement a program model that combined group health education classes and telephone and in-home counseling. The workgroup also determined the length, frequency, and dosage of the program.

Bilingual health educators who were employed by the local county hospital system led the program due to their established working relationships with the facilities. Thirteen bilingual pharmacists were recruited from a local network of university pharmacy student preceptors. The pharmacists completed 20 hours of motivational interviewing training conducted by the health educators and online health literacy, hypertension, and diabetes management training. Both pharmacists and health educators were fluent in Spanish, Vietnamese, Mandarin, or Cantonese.

The 12-week intervention included a pharmacist-led home visit and two telephone consultations. Pharmacists were assigned participants based on the shared languages spoken. The one-hour home visit involved a comprehensive medication review and a medication list for the patient to keep. Participants were provided a pillbox that could be used during the session to demonstrate how to sort their medication for the week and a pill cutter for use when needed. Telephone follow-up calls occurred two weeks after each group health education session.

The health educators provided two sessions using culturally tailored printed materials. The curriculum for the “Healthy Eating” session was adapted from the Myplate.gov nutrition education series. Participants were taught the benefits of eating healthy, balancing calories, portion control, how to read food labels, and how to tailor their diet to their chronic disease. In the “Being Active and Managing Stress” session, participants received an overview of benefits and recommended exercises.

Small incentives were provided throughout the program. After the program, participants were provided a $15 gift card to a local store to purchase groceries or home goods as an incentive for participation.

## Results

MyRx staff facilitated focus groups at each facility following the program’s completion. A 13-item questionnaire was used to guide the focus group discussions. Themes captured through the questionnaire focused on the participants’ view of the program’s in-home setting, program delivery method, program satisfaction, and behavioral intentions to adopt a healthy lifestyle.

A total of 51 participants enrolled in the program, and 38 completed the program for an attrition rate of 25%. Table 1 describes the diversity of the participants enrolled in the program. Table 2 summarizes the most salient themes of the focus groups.

## Discussion

Overall, seniors were satisfied with the program, had an appreciation for individual counseling, and were likely to change behavior with ongoing interactions with a health care professional. This program integrates pharmacists and health educator teams to improve patient care. Research indicates the value of interdisciplinary approaches to chronic disease state management [11,12]. This multidisciplinary approach is the basis for the PCMH model promoted by the Agency for Healthcare Research and Quality for utilization in the primary care setting to deliver comprehensive, patient-centered care and have coordinated care and safe and accessible services quality [6]. This program’s strength is it provides services to a diverse population in four diff erent languages individually tailored to each participant’s cultural background. Limitations of this study include the short intervention period and small sample size. A longer longitudinal service will provide more information about the long-term impact on clinical outcomes.

## Conclusion

Qualitative analysis of focus group discussions revealed participant satisfaction with the MY Rx program and willingness to change as a result of participation in the program. Future studies should be conducted over a more extended time to determine pharmacists and health educators’ clinical impact. Additional recommendations include further analysis of the professional team dynamics and analysis of participants’ satisfaction with processes used to engage them in planning.

## Figures and Tables

**Table 1:** Characteristics of MY Rx Participants at Baseline.

Variable	Total N=51 N (%)	Facility A N=26 N (%)	Facility B N=6 N (%)	Facility C N=18 N (%)	Facility D N=1 N (%)
Race (n=51)*					
African American	25 (49)	16 (61.5)	3 (50)	5 (27.8)	1 (100)
Asian American	14 (27)	3 (11.5)	0 (0)	11 (61.1)	0 (0)
White	3 (6)	2 (7.7)	0 (0)	1 (5.6)	0 (0)
Other/Missing	9 (18)	5 (19.2)	3 (50)	1 (5.6)	0 (0)
Ethnicity (n=51)*					
Hispanic/Latino	13 (25)	6 (23)	1 (17)	6 (33)	0 (0)
Non-Hispanic/Latino	38 (75)	20 (77)	5 (83)	12 (77)	1 (100)
Sex (n=51)*					
Male	30 (59)	22 (85)	1 (17)	7 (39)	0 (0)
Female	21 (41)	4 (15)	5 (83)	11 (61)	1 (100)
Age (n=51)*					
Greater than 55 years	50 (98)	26 (100)	6 (100)	17 (94)	1 (100)
Primary Language (n=51)*					
English	35 (68.6)	21 (80.8)	5 (83)	8 (44)	1 (100)
Mandarin	10 (19.2)	1 (3.9)	0 (0)	9 (50)	0 (0)
Spanish	5 (9.8)	3 (11.5)	1 (17)	1 (6)	0 (0)
Vietnamese	1 (2)	1 (3.9)	0 (0)	0 (0)	0 (0)
Education Level (n=47)*					
Middle school or lower	4 (8.5)	4 (16)	0 (0)	0 (0)	0 (0)
School certificate	2 (4.3)	2 (8)	0 (0)	0 (0)	0 (0)
High school graduate	29 (61.7)	14 (56)	5 (100)	9 (56)	1 (100)
Some college	3 (6.4)	2 (8)	0 (0)	1 (6)	0 (0)
College graduate	5 (10.6)	3 (12)	0 (0)	2 (13)	0 (0)
More than 16 years	4 (8.5)	0 (0)	0 (0)	4 (25)	0 (0)

* number of participants responded to question

**Table 2:** Thematic Analysis of Qualitative Data (N=22).

Theme	Participants’ Quotes

Participants were satisfied with the program.	*“I participated in this program right from the beginning and I learn so many things. I’m very, very happy. It gives us a lot of knowledge; it gives us a lot of help.”*
	*“It’s a good program, and if you have it again next year, I’ll be right in. I enjoyed it.”*

Participants shared the best methods used by the program to deliver information and services.	*“One-on-one, I liked that. She (pharmacist) used her time wisely.”*
	*“I like the one-on-one contact both on the phone and at the apartment.”*
	*“It (pharmacist’s phone call) was like a reminder, if that person would’ve forgotten it (taking medication) that morning.”*

Participants reported that participation in the program improved their medication adherence.	*“The program helped me because every now and then I let my sugar control me, instead of me controlling it.”*
	*“I know when I (was) first contacted, the problem that I had, I didn’t want to take my medication. I had to learn the hard way. They’re helping me; I’m not helping myself by not taking it. The medicine is good you have to do what your doctor tells you.”*
	*“I used to drink a lot of alcohol. The medication would not work with the alcohol. I had to stop drinking. The main thing was the alcohol and the medicine.”*

## Discover a bigger Impact and Visibility of your article publication with Peertechz Publications

### Highlights

- Signatory publisher of ORCID
- Signatory Publisher of DORA (San Francisco Declaration on Research Assessment)
- Articles archived in worlds' renowned service providers such as Portico, CNKI, AGRIS, TDNet, Base (Bielefeld University Library), CrossRef, Scilit, J-Gate etc.
- Journals indexed in ICMJE, SHERPA/ROMEO, Google Scholar etc.
- OAI-PMH (Open Archives Initiative Protocol for Metadata Harvesting)
- Dedicated Editorial Board for every journal
- Accurate and rapid peer-review process
- Increased citations of published articles through promotions
- Reduced timeline for article publication
